# Supplementary material for: Heat adaptation of phage T7 under an extended genetic code
Source: Virus Evol. 2021 Dec 1;7(2):veab100. doi: 10.1093/ve/veab100 (PMC8923235; doi:10.1093/ve/veab100)
Supplement: veab100_Supp [file veab100_supp.zip › Sequencing_Analysis.zip!/Sequencing_Analysis/S2/evidence/SNP_3.html]

BRESEQ :: Evidence


| Predicted mutation | | | | | | |
| --- | --- | --- | --- | --- | --- | --- |
| evidence | seq id | position | mutation | annotation | gene | description |
| RA | NC\_001604.1 | 15,094 | G→T | intergenic (–/–) | – / – | –/– |

| Read alignment evidence... | | | | | | | | | | | |
| --- | --- | --- | --- | --- | --- | --- | --- | --- | --- | --- | --- |
|  | seq id | position | | ref | new | freq | score (cons/poly) | reads | annotation | genes | product |
| \* | NC\_001604.1 | 15,094 | 0 | G | T | 99.6% | 14049.6 / NA | 5484 | intergenic (–/–) | *–/–* | –/– |
| Reads supporting (aligned to +/- strand):  **ref** base G (1/21);  **new** base T (2729/2715);  **total** (2739/2745) | | | | | | | | | | | |
| Fisher's exact test for biased strand distribution *p*-value = 1.06e-05 | | | | | | | | | | | |
| Kolmogorov-Smirnov test that lower quality scores support variant *p*-value = 1.00e+00 | | | | | | | | | | | |

Only 100 of 5502 total aligned reads displayed.  

`GAGCAGGGTGAAGAATACGTTGACGGAATGGAGTGGTGGAACTTCAACGAAGAGATGATGGACTATAACGTTCAGGACGTTGTGGTAACTAAAGCTCTCCTTGAGAAGCTACTCTCTGACAAACATTACTTCCCTCCTGAGATTGACTTTACGGACGTAGGATACACTACGTTCTGGTCAGAAT‑CCCTTGAGGCCGTTGACATTGAACATCGTGCTGCATGGCTGCTCGCTAAACAAGAGCGCAACGGGTTCCCGTTTGACACAAAAGCAATCGAAGAGTTGTACGTAGAGTTAGCTGCTCGCCGCTCTGAGTTGCTCCGTAAATTGACCGAAACGTTCGGCTCGTGGTATCAGCCTAAAGGTGGCACTGAGATGTTCTGCCATCCGCGAACAGGTAAGCCACTACCTAAATACCCTCGCATTAAGACACCTAAAGTTGGTGGTATCTTTAAGAAGCCTAAGAACAAGGCACAGCGAGAAGGCCGTGAGCCTTGCGAACTTGATACCCGCGAGTACGTTGCTGGTGCTCCTTACACCCCAGTTGAACATGTTGTGTTTAACCCTTCGTCTCGTGACCACATTCAGAAGAAACTCC  >  NC_001604.1/14797‑15391`  

                                                                                                                                                                                                                                                                                                          `|`                                                                                                                                                                                                                                                                                                           
    `aGGGTGAAGAATACGTTGACGGAATGGAGTGGTGGAACTTCAACGAAGAGATGATGGACTATAACGTTCAGGACGTTGTGGTAACTAAAGCTCTCCTTGAGAAGCTACTCTCTGACAAACATTACTTCCCTCCTGAGATTGACTTTACGGACGTAGGATACACTACGTTCTGGTCAGAAT‑CCCTTGAGGCCGTTGACATTGAACATCGTGCTGCATGGCTGCTCGCTAAACAAGAGCGCAACGGGTTCCCGTTTGACACAAAAGCAATCGAAGAGTTGTACGTAGAGTTAGCTTCTcgccg                                                                                                                                                                                                                                                                                                    >  1:23414/1‑301 (MQ=255)`  
     `gggTGAAGAATACGTTGACGGAATGGAGTGGTGGAACTTCAACGAAGAGATGATGGACTATAACGTTCAGGACGTTGTGGTAACTAAAGCTCTCCTTGAGAAGCTACTCTCTGACAAACATTACTTCCCTCCTGAGATTGACTTTACGGACGTAGGATACACTACGTTCTGGTCAGAAT‑CCCTTGAGGCCGTTGACATTGAACATCGTGCTGCATGGCTGCTCGCTAAACAAGAGCGCAACGGGTTCCCGTTTGACACAAAAGCAATCGAAGAGTTGTACGTAGAGTTAGCTTCTcgccgc                                                                                                                                                                                                                                                                                                   >  1:626466/1‑301 (MQ=255)`  
     `gggTGAAGAATACGTTGACGGAATGGAGTGGTGGAACTTCAACGAAGAGATGATGGACTATAACGTTCAGGACGTTGTGGTAACTAAAGCTCTCCTTGAGAAGCTACTCCCTGACAAACATTACTTCCCTCCTGAGATTGACTTTACGGACGTAGGATACACTACGTTCTGGTCAGAAT‑CCCTTGAGGCTGTTGACATTGAACATCGTGCTGCATGGCTGTTCGCTAAACACGAGCGCAACGGGTTCCCGTTCGACACAAAAGCAAACGAAGTGTTGTTCGTAGAGTTAGCTTCTcgccgc                                                                                                                                                                                                                                                                                                   >  1:274774/1‑301 (MQ=255)`  
       `taagagacagcGTTGACGGAATGTAGTGGTGGAACTTCAACGAAGAGATGATGGACTATAACGTTCAGGACGTTGTGGTAACTAAAGCTCTCCTTGAGAAGCTACTCTCTGACAAACATTACTTCCCTCCTGAGATTGACTTTACGGACGTAGGATACACTACGTTCTGGTCAGAAT‑CCCTTGAGGCCGTTGACATTGAACATCGTGCTGCATGGCTGCTCGCTAAACAAGAGCGCAACGGGTTCCCGTTTGACACAAAAGCAATCGAAGAGTTGTACGTAGAGTTAGCTTCTCGCCGctc                                                                                                                                                                                                                                                                                                 <  1:38694/291‑1 (MQ=255)`  
       `gTGAAGAATACGTTGACGGAATGGAGTGGTGGAACTTCAACGAAGAGATGATGGACTATAACGTTCATGACGTTGTGGTAACTAAAGCTCTCCTTGAGAAGCTACTCTCTGACAAACATTACTTCCCTCCTGAGATTGACTTTACGGACGTAGGATACACTACGTTCTGGTCAGAAT‑CCCTTGAGGCCGTTGACATTGAACATCGTGCTGCATGGCTGCTCGCTAAACATGAGCGCAACGGGTTCCCGTTTGACACAAAAGCAATCGAAGAGTTGTACGTAGAGTTAGCTTCTCGCCCctc                                                                                                                                                                                                                                                                                                 >  1:443002/1‑301 (MQ=255)`  
           `agaaTACGTTGACGGAATGGAGTGGTGGAACTTCAACGAAGAGATGATGGACTATAACGTTCAGGACGTTGTGGTAACTAAAGCTCTCCTTGAGAAGCTACTCTCTGACAAACATTACTTCCCTCCTGAGATTGACTTTACGGACGTAGGATACACTACGTTCTGGTCAGAAT‑CCCTTGAGGCCGTTGACATTGAACATCGTGCTGCATGGCTGCTCGCTAAACAAGAGCGCAACGGGTTCCCGTTTGACACAAAAGCAATCGAAGAGTTGTACGTAGAGTTAGCTTCTCGCCGCTCTGAg                                                                                                                                                                                                                                                                                             >  1:75153/1‑301 (MQ=255)`  
           `agaaTACGTTGACGGAATGGAGTGGTGGAACTTCAACGAAGAGATGATGGACTATAACGTTCAGGACGTTGTGGTAACTAAAGCTCTCCTTGAGAAGCTACTCTCTGACAAACATTACTTCCCTCCTGAGATTGACTTTACGGACGTAGGATACACTACGTTCTGGTCAGAAT‑CCCTTGAGGCCGTTGACATTGAACATCGTGCTGCATGGCTGCTCGCTAAACAAGAGCGCAACGGGTTCCCGTTTGACACAAAAGCAATCGAAGAGTTGTACGTAGAGTTAGCTTCTCGCCGCTCTGAg                                                                                                                                                                                                                                                                                             >  1:5959/1‑301 (MQ=255)`  
            `aaaTACGTTGACGGAATGGAGTGGGGGAACTTCAACGAAGAGATGATGGACTATAACGTTCAGGACGTTGTGGTAACTAAAGCTCTCCTTGAGAAGCTACTCTCTGACAAACATTACTTCCCTCCTGAGATTGACTTTACGGACGTAGGATACACTACGTTCTGGTCAGAAT‑CCCTTGAGGCCGTTGACATTGAACATCGTGCTGCATGGCTGCTCGCTAAACAAGAGCGCAACGGGTTCCCGTTTGACACAAAAGCAATCGAAGAGTTGTACGTAGAGTTAGCTTCTCGCCGCTCTGAGt                                                                                                                                                                                                                                                                                            <  1:318663/300‑1 (MQ=255)`  
                 `cGTTGACGGAATGGAGTGGTGGAACTTCAACGAAGAGATGATGGACTATAACGTTCAGGACGTTGTGGTAACTAAAGCTCTCCTTGAGAAGCTACTCTCTGACAAACATTACTTCCCTCCTGAGATTGACTTTACGGACGTAGGATACACTACGTTCTGGTCAGAAT‑CCCTTGAGGCCGTTGACATTGAACATCGTGCTGCATGGCTGCTCGCTAAACAAGAGCGCAACGGGTTCCCGTTTGACACAAAAGCAATCGAAGAGTTGTACGTAGAGTTAGCTTCTCGCCGCGCTGAGTTGCTc                                                                                                                                                                                                                                                                                       >  1:174062/1‑301 (MQ=255)`  
                 `cGTTGACGGAATGGAGTGGGGGAACTTCAACGAAGAGATGATGGACTATAACGTTCAGGACGTTGTGGTAACTAAAGCTCTCCTTGAGAAGCTACTCTCTGACAAACATTACTTCCCTCCTGAGATTGACTTTACGGACGTAGGATACACTACGTTCTGGTCAGAAT‑CCCTTGAGGACGTTGACATTGAACATCGTGCTGCATGGCTGCTCGCTAAACAAGAGCGCAACGGGTTCCCGTTTGACACAAAAGCAATCGAAGAGTTGTACGTAGAGTTAGCTTCTCGCCGCTCTGAGTTGCTc                                                                                                                                                                                                                                                                                       <  1:480916/301‑1 (MQ=255)`  
                      `aCGGAATGGAGTGGTGGAACTTCAACGAAGAGATGATGGACTATAACGTTCAGGACGTTGTGGTAACTAAAGCTCTCCTTGAGAAGCTACTCTCTGACAAACATTACTTCCCTCCTGAGATTGACTTTACGGACGTAGGATACACTACGTTCTGGTCAGAAT‑CCCTTGAGGCCGTTGACATTGAACATCGTGCTGCATGGCTGCTCGCTAAACAAGAGCGCAACGGGTTCCCGTTTGACACAAAAGCAATCGAAGAGTTGTACGTAGAGTTAGCTTCTCGCCGCTCTGAGTTGCTCCGTaa                                                                                                                                                                                                                                                                                  >  1:714071/1‑301 (MQ=255)`  
                       `cGGAATGGAGTGGTGGAACTTCAACGAAGAGATGATGGACTAAAACGTTCAGGACGTTGTGGTAACTAAAGCTCTCCTTGAGAAGCTACTCTCTGACAAACATTACTTCCCTCCTGAGATTGACTTTACGGACGTAGGATACACTACGTTCTGGTCAGAAT‑CCCTTGAGGCCGTTGACATTGAACATCGTGCTGCATGGCTGCTCGCTAAACAAGAGCGCAACGGGTTCCCGTTTGACACAAAAGCAATCGAAGAGTTGTACGTAGAGTTAGCTTCTCGCCGCTCAGAGTTGCTCCGTaaa                                                                                                                                                                                                                                                                                 <  1:290015/301‑1 (MQ=255)`  
                        `ggAATGGAGTGGTGGAACTTCAACGAAGAGATGATGGACTATAACGTTCAGGACGTTGTGGTAACTAAAGCTCTCCTTGAGAAGCTACTCTCTGACAAACATTACTTCCCTCCTGAGATTGACTTTACGGACGTAGGATACACTACGTTCTGGTCAGAAT‑CCCTTGAGGCCGTTGACATTGAACATCGTGCTGCATGGCTGCTCGCTAAACAAGAGCGCAACGGGTGCCCGTTTGACACAAAAGCAATCGAAGAGTTGTACGTAGAGTTAGCTTCTCGCCGCTCTGAGTTGCTCCGTACAt                                                                                                                                                                                                                                                                                >  1:152846/1‑301 (MQ=255)`  
                        `cgtgggctcggagatgtgtataagagacagaTGATGGACTATAACGTTCAGGACGTTGTGGTAACTAAAGCTCTCCTTGAGAAGCTACTCTCTGACAAACATTACTTCCCTCCTGAGATTGACTTTACGGACGTAGGATACACTACGTTCTGGTCAGAAT‑CCCTTGAGGCCGTTGACATTGAACATCGTGCTGCATGGCTGCTCGCTAAACAAGAGCGCAACGGGTTCCCGTTTGACACAAAAGCAATCGAAGAGTTGTACGTAGAGTTAGCTTCTCGCCGCTCTGAGTTGCTCCGTAAAt                                                                                                                                                                                                                                                                                <  1:728482/273‑1 (MQ=255)`  
                         `gAATGGAGTGGTGGAACTTCAACGAAGAGATGATGGACTATAACGTTAAGGACGTTGTGGTAACTAAAGCTCTCCTTGAGAAGCTACTCTCTGACAAACATTACTTCCCTCCTGAGATTGACTTTACGGACGTAGGATACACTACGTTCTGGTCAGAAT‑CCCTTGAGGCCGTTGACATTGAACATCGTGCTGCGTGGCTGCTCGCTAAACAAGAGCGCAACGGGTTCCCGTTTGACACAAAAGCAATCGAAGAGTTGTACGTAGAGTTAGCTTCTCGCCGCTCTGAGTTGCTCCGTAAAtt                                                                                                                                                                                                                                                                               <  1:640440/301‑1 (MQ=255)`  
                             `gacagggtggAACTTCAACGAAGAGATGATGGACTATAACGTTCAGGACGTTGTGGTAACTAAAGCTCTCCTTGAGAAGCTACTCTCTGACAAACATTACTTCCCTCCTGAGATTGACTTTACGGACGTAGGATACACTACGTTCTGGTCAGAAT‑CCCTTGAGGCCGTTGACATTGAACATCGTGCTGCATGGCTGCTCGCTAAACAAGAGCGCAACGGGTTCCCGTTTGACACAAAAGCAATCGAAGAGTTGTACGTAGAGTTAGCTTCTCGCCGCTCTGAGTTGCTCCGTAAATTGAcc                                                                                                                                                                                                                                                                           <  1:669310/296‑1 (MQ=255)`  
                                           `tgtgtataagagacagggACTATAACGTTCAGGACGTTGTGGTAACTAAAGCTCTCCTTGAGAAGCTACTCTCTGACAAACATTACTTCCCTCCTGAGATTGACTTTACGGACGTAGGATACACTACGTTCTGGTCAGAAT‑CCCTTGAGGCCGTTGACATTGAACATCGTGCTGCATGGCTGCTCGCTAAACAAGAGCGCAACGGGTTCCCGTTTGACACAAAAGCAATCGAAGAGTTGTACGTAGAGTTAGCTTCTCGCCGCTCTGAGTTGCTCCGTAAATTGACCGAAACGTTCGGCTc                                                                                                                                                                                                                                                             <  1:312418/285‑1 (MQ=255)`  
                                           `tCAACGAAGAGATGATGGACTATAACGTTCAGGACGTTGTGGTAACTAAAGCTCTCCTTGAGAAGCTACTCTCTGACAAACATTACTTCCCTCCTGAGATTGACTTTACGGACGTAGGATACACTACGTTCTGGTCAGAAT‑CCCTTGAGGCCGTTGACATTGAACATCGTGCTGCATGGCTGCTCGCTAAACAAGAGCGCAACGGGTTCCCGTTTGACACAAAAGCAATCGAAGAGTTGTACGTAGAGTTAGCTTCTCGCCGCTCTGAGTTGCTCCGTAAATTGACCGAAACGTTCGGCTc                                                                                                                                                                                                                                                             >  1:550629/1‑301 (MQ=255)`  
                                                   `gagcTGATGGACTATAACGTTCAGGACGTTGTGGTAACTAAAGCTCTCCTTGAGAAGCTACTCTCTGACAAACATTACTTCCCTCCTGAGATTGACTTTACGGACGTAGGATACACTACGTTCTGGTCAGAAT‑CCCTTGAGGCCGTTGACATTGAACATCGTGCTGCATGGCTGCTCGCTAAACAAGAGCGCAACGGGTTCCCGTTTGACACAAAAGCAATCGAAGAGTTGTACGTAGAGTTAGCTTCTCGCCGCTCTGAGTTGCTCCGTAAATTGACCGAAACGTTCTGCTCGTGGTATc                                                                                                                                                                                                                                                     >  1:735887/1‑301 (MQ=255)`  
                                                   `gagaTGATGGACTATAACGTTCAGGACGTTGTGGTAACTAAAGCTCTCCTTGAGAAGCTACTCTCTGACAAACATTACTTCCCTCCGGAGATTGACTTTACGGACGTAGGATACACTACGTTCTGGTCAGAAT‑CCCTTGAGGCCGTTGACATTGAACATCGTGCTGCATGGCTGCTCGCTAAACAAGAGCGCAACGGGTTCCCGTTTGACACAAAAGCAATCGAAGAGTTGTACGTAGAGTTAGCTTCTCGCCGCTCTGAGTTGCTCCGTAAATTGACCGAAACGTTCGGCTCGTGGTATc                                                                                                                                                                                                                                                     <  1:69082/301‑1 (MQ=255)`  
                                                   `cagaTGATGGACTATAACGTTCAGGACGTTGTGGTAACTAAAGCTCTCCTTGAGAAGCTACTCTCTGATAAACATTACTTCCCTCCTGAGATTGACTTTACGGACGTAGGATACACTACGTTCTGGTCAGAAT‑CCCTTGAGGCCGTTGACATTGAACATCGTGCTGCATGGCTGCTCGCTAAACAAGAGCGCAACGGGTTCCCGTTTGACACAAAAGCAATCGAAGAGTTGTACGTAGAGTTAGCTTCTCGCCGCTCTGAGTTGCTCCGTAAATTGACCGAAACGTTCGGCTCGTGGTATc                                                                                                                                                                                                                                                     <  1:697681/300‑1 (MQ=255)`  
                                                       `tgatgTACTATAATGTTCAGGACGTTGTGGTAACTAAAGCTCTCCTTGAGAAGCTACTCTCTGACAAACATTACTTCCCTCCTGAGATTGACTTTACGGACGTAGGATACACTACGTTCTGGTCAGAAT‑CCCTTGAGGCCGTTGACATTGAACATCGTGCTGCATGGCTGCTCGCTAAACAAGAGCGCAACGGGTTCCCGTTTGACACAAAAGCAATCGAAGAGTTGTACGTAGAGTTAGCTTCTCGCCGCTCTGAGTTGCTCCGTAAATTGACCGAAACGTTCGGCTCGTGGTATCAGcc                                                                                                                                                                                                                                                 <  1:435545/301‑1 (MQ=255)`  
                                                       `tgatgGGCTATAACGTTCAGGACGTTGTGGTAACTAAAGCTCTCCTTGAGAAGCTACTCTCTGACAAACATTACTTCCCTCCTGAGATTGACTTTACGGACGTAGGATACACTACGTTCTGGTCAGAAT‑CCCTTGAGGCCGTTGACATTGAACATCGTGCTGCATGGCTGCTCGCTAAACAAGAGCGCAACGGGTTCCCGTTTGACACAAAAGCAATCGAAGAGTTGTACGTAGAGTTAGCTTCTCGCCGCTCTGAGTTGCTCCGTAAATTGACCGAAACGTTCGGCTCGTGGTATCAGcc                                                                                                                                                                                                                                                 <  1:11543/301‑1 (MQ=255)`  
                                                       `acagggACTATAACGTTCAGGACGTTGTGGTAACTAAAGCTCTCCTTGAGAAGCTACTCTCTGACAAACATTACTTCCCTCCTGAGATTGACTTTACGGACGTAGGATACACTACGTTCTGGTCAGAAT‑CCCTTGAGGCCGTTGACATTGAACATCGTGCTGCATGGCTGCTCGCTAAACAAGAGCGCAACGGGTTCCCGTTTGACACAAAAGCAATCGAAGAGTTGTACGTAGAGTTAGCTTCTCGCCGCTCTGAGTTGCTCCGTAAATTGACCGAAACGTTCGGCTCGTGGTATCAGcc                                                                                                                                                                                                                                                 <  1:661983/297‑1 (MQ=255)`  
                                                           `ggACTATAACGTTCAGGACGTTGTGGTAACTAAAGCTCTCCTTGAGAAACTACTCTCTGACAAACATTACTTCCCTCCTGAGATTGACTTTACGGACGTAGGATACACTACGTTCTGGTCAGAAT‑CCCTTGAGGCCGTTGACATTGAACATCGTGCTGCATGGCTGCTCGCTAAACAAGAGCGAAACGGGTTCCCGTTTGACACAAAAGCAATCGAAGAGTTGTACGTAGAGTTAGCTTCTCGCCGCTCTGAGTTGCTCCGTAAATTGACCGAAACGTTCGGCCCGTGGTATCAGCCTaaa                                                                                                                                                                                                                                             >  1:108275/1‑301 (MQ=255)`  
                                                             `gcTATAACGTTCAGGACGTTGTGGTAACTAAAGCTCTCCTTGAGAAGCTACTCTCTGACAAACATTACTTCCCTCCTGAGATTGACTTTACGGACGTAGGATACACTACGTTCTGGTCAGAAT‑CCCTTGAGGCCGTTGACATTGAACATCGTGCTGCATGGCTGCTCGCTAAACAAGAGCGCAACGGGTTCCCGTTTGACACAAAAGCAATCGAAGAGTTGTACGTAGAGTTAGCTTCTCGCCGCTCTGAGTTGCTCCGTAAATTGACCGAAACGTTCGGCTCGTGGTATCAGCCTAAAgg                                                                                                                                                                                                                                           <  1:466049/300‑1 (MQ=255)`  
                                                             `ccTATTACGTTCAGGACGGTGTGGTAACTAAAGCTCTCCTTGAGAAGCTACTCTCTGACAAACATTACTTCCCTCCTGAGATTGACTTTACGGACGTAGGATACACTACGTTCTGGTCAGAAT‑CCCTTGAGGCCGTTGACATTGAACATCGTGCTGCATGGCTGCTCGCTAAACAAGAGCGCAACGGGTTCCCGTTTGACACAAAAGCAATCGAAGAGTTGTACGTAGAGTTAGCTTCTCGCCGCTCTGAGTTGCTCCGTAAATTGACCGAAACGTTCGGCTCGTGGTATCAGCCTAAAgg                                                                                                                                                                                                                                           <  1:22703/300‑1 (MQ=255)`  
                                                             `agacagaCGTTCAGGACGTTGTGGTAACTAAAGCTCTCCTTGAGAAGCTACTCTCTGACAAACATTACTTCCCTCCTGAGATTGACTTTACGGACGTAGGATACACTACGTTCTGGTCAGAAT‑CCCTTGAGGCCGTTGACATTGAACATCGTGCTGCATGGCTGCTCGCTAAACAAGAGCGCAACGGGTTCCCGTTTGACACAAAAGCAATCGAAGAGTTGTACGTAGAGTTAGCTTCTCGCCGCTCTGAGTTGCTCCGTAAATTGACCGAAACGTTCGGCTCGTGGTATCAGCCTAAAgg                                                                                                                                                                                                                                           <  1:450544/295‑1 (MQ=255)`  
                                                             `aagataACGTTCAGGACGTTGTGGTAACTAAAGCTCTCCTTGAGAAGCTACTCTCTGACAAACATTACTTCCCTCCTGAGATTGACTTTACGGACGTAGGATACACTACGTTCTGGTCAGAAT‑CCCTTGAGGCCGTTGACATTGAACATCGTGCTGCATGGCTGCTCGCTAAACAAGAGCGCAACGGGTTCCCGTTTGACACAAAAGCAATCGAAGAGTTGTACGTAGAGTTAGCTTCTCGCCGCTCTGAGTTGCTCCGTAAATTGACCGAAACGTTCGGCTCGTGGTATCAGCCTAAAgg                                                                                                                                                                                                                                           <  1:633040/298‑1 (MQ=255)`  
                                                             `aCTATAACGTTCAGGACGTTGTGGTAACTAAAGCTCTCCTTGGGAAGCTACTCTCTGACAAACATTACTTCCCTCCTGAGATTGACTTTACGGACGTAGGATACACTACGTTCTGGTCAGAAT‑CCCTTGAGGCCGTTGACATTGAACATCGTGCTGCATGGCTGCTCGCTAAACAAGAGCGCAACGGGTTCCCGTTTGACACAAAAGCAATCGAAGAGTTGTACGTAGAGTTAGCTTCTCGCCGCTCTGAGTTGCTCCGTAAATTGACCGAAACGTTCGGCTCGTGGTATCAGCCTAAAgg                                                                                                                                                                                                                                           <  1:564789/301‑1 (MQ=255)`  
                                                             `aCTATAACGTTCAGGACGTTGTGGTAACTAAAGCTCTCCTTGAGAAGCTACTCTCTTACAAACATTACTTCCCTCCTGAGATTGACTTTACGGACGTAGGATACACTACGTTCTGGTCAGAAT‑CCCTTGAGGCCGTTGACATTGAACATCGTGCTGCATGGCTGCTCGCTAAACAAGAGCGCAACGGGTTCCCGTTTGACACAAAAGCAATCGAAGAGTTGTACGTAGAGTTAGCTTCTCGCCGCTCTGAGTTGCTCCGTAAATTGACCGAAACGTTCGGCTCGTGGTATCAGCCTAAAgg                                                                                                                                                                                                                                           <  1:123667/301‑1 (MQ=255)`  
                                                             `aCTATAACGTTCAGGACGTTGTGGTAACTAAAGCTCTCCTTGAGAAGCTACTCTCTGACAAACATTTCTTCCCTCCTGAGATTGACTTTACGGACGTAGGATACACTACGTTCTGGTCAGAAT‑CCCTTGAGGCCGTTGACATTGAACATCGTGCTGCATGGCTGCTCGCTAAACAAGAGCGAAACGGGTTCCCGTTTGACACAAAAGCAATCGAAGAGTTGTACGTAGAGTTAGCTTCTCGCCGCTCTGAGTTGCTCCGTAAATTGACCGAAACGTTCGGCTCGTGGTATCAGCCTAAAgg                                                                                                                                                                                                                                           <  1:705191/301‑1 (MQ=255)`  
                                                             `aCTATAACGTTCAGGACGTTGTGGTAACTAAAGCTCTCCTTGAGAAGCTACTCTCTGACAAACATTACTTCCCTCCTGAGATTGACTTTACGGACGTAGGATACACTACGTTCTGGTCAGAAT‑CCCTTGAGGCCGTTGACATTGAACATCGTGCTGCATGGCTGCTCGCTAAACAAGAGCGCAACGGGTTCCCGTTTGACACAAAAGCAATCGAAGAGTTGTACGTAGAGTTAGCTTCTCGCCGCTCTGAGTTGCTCCGTACATTGACCGAAACGGTCGGCTCGTGGTATCAGCCTAAAgg                                                                                                                                                                                                                                           >  1:100000/1‑301 (MQ=255)`  
                                                             `aCTATAACGTTCAGGACGTTGTGGTAACTAAAGCTCTCCTTGAGAAGCTACTCTCTGACAAACATTACTTCCCTCCTGAGATTGACTTTACGGACGTAGGATACACTACGTTCTGGTCAGAAT‑CCCTTGAGGCCGTTGACATTGAACATCGTGCTGCATGGCTGCTCGCTAAACAAGAGCGCAACGGGTTCCCGTTTGACACAAAAGCAATCGAAGAGTTGTACGTAGAGTTAGCTTCTCGCCGCTCTGAGTTGCTCCGTAAATTGACCGAAACGTTCGGCTCGTGGTATCAGCCTAAAgg                                                                                                                                                                                                                                           <  1:758466/301‑1 (MQ=255)`  
                                                                 `taACGTTCAGGACGTTGTGGTAACTAAAGCTCTCCTTGAGAAGCTACTCTCTGACAAACATTACTTCCCTCCTGAGATTGACTTTACGGACGTAGGATACACTACGTTCTGGTCAGAAT‑CCCTTGAGGCCGTTGACATTGAACATCGTGCTGCATGGCTGCTCGCTAAACAAGAGCGCAACGGGTTCCCGTTTGACACAAAAGCAATCGAAGAGTTGTACGTAGAGTTAGCTTCTCGCCGCTCTGAGTTGCTCCGTAAATTGACCGAAACGTTCGGCTCGTGGTATCAGCCTAAAGGTGGc                                                                                                                                                                                                                                       <  1:50769/301‑1 (MQ=255)`  
                                                                   `aCGTTCAGGACGTTGTGGTAACTAAAGCTCTCCTTGAGAAGCTACTCTCTGACAAACATTACTTCCCTCCTGAGATTGACTTTACGGACGTAGGATACACTACGTTCTGGTCAGAAT‑CCCTTGAGGCCGTTGACATTGAACATCGTGCTGCATGGCTGCTCGCTAAACAAGAGCGCAACGGGTTCCCGTTTGACACAAAAGCAATCGAAGAGTTGTACGTAGAGTTAGCTTCTCGCCTCTCTGAGTTGCTCCGTAAATTGACCGAAACGTTCGGCTCGTGGTTTCAGCCTACAGGTGGCAc                                                                                                                                                                                                                                     >  1:498990/1‑301 (MQ=255)`  
                                                                    `cGTTCAGGACGTTGTGGTAACTAAAGCTCTCCTTGAGAAGCTACTCTCTGACAAACATTACTTCCCTCCTGAGATTGACTTTACGGACGTAGGATACACTACGTTCTGGTCAGAAT‑CCCTTGAGGCCGTTGACATTGAACATCGTGCTGCATGGCTGCTCGCTAAACAAGAGCGCAACGGGTTCCCGTTTGACACAAAAGCAATCGAAGAGTTGTACGTAGAGTTAGCTTCTCGCCGCTCTGATTTGCTCCGTAAATTGACCGAAACGTTCGGCTCGTGGTATCAGCCTAAAGGTGGCACt                                                                                                                                                                                                                                    >  1:558366/1‑301 (MQ=255)`  
                                                                        `gaggagtttGTGGTAACTAAAGCTCTCCTTGAGAAGCTACTCTCTGACAAACATTACTTCCCTCCTGAGATTGACTTTACGGACGTAGGATACACTACGTTCTGGTCAGAAT‑CCCTTGAGGCCGTTGACATTGAACATCGTGCTGCATGGCTGCTCGCTAAACAAGATCGCAACGGGTTCCCGTTTGACACAAAAGCAATCGAAGAGTTGTACGTAGAGTTAGCTTCTCGCCGCTCTGAGTTGCTCCGTAAATTGACCGAACCGTTCGGCTCGTGGTATCAGCCTAAAGGTGGCACTgaga                                                                                                                                                                                                                                <  1:619686/294‑1 (MQ=255)`  
                                                                        `acagACGTTGTGGTAACTAAAGCTCTCCTTGAGAAGCTACTCTCTGACAAACATTACTTCCCTCCTGAGATTGACTTTACGGACGTAGGATACACTACGTTCTGGTCAGAAT‑CCCTTGAGGCCGTTGACATTGAACATCGTGCTGCATGGCGGCTCGCTAAACAAGAGCGCAACGGGTTCCCGTTTGACACAAAAGCAATCGAAGAGTTGTACGTAGAGTTAGCTTCTCGCCGCTCTGAGTTGCTCCGTAAATTGACCGAAACGTTCGGCTCGTGGTATCAGCCTAAAGGTGGCACTgaga                                                                                                                                                                                                                                <  1:536493/298‑1 (MQ=255)`  
                                                                           `aaCGTAGTGGTAACTAAAGCTCTCCTTGAGAAGCTACTCTCTGACAAACATTACTTCCCTCCTGAGATTGACTTAACGGACGTAGGATACACTACGTTCTGGTCAGAAT‑CCCTGGAGGCCGTTGACATTGAACATCGTGCTGCATGGCTGCTCGCTAAACAAGAGCGAAACGGGTTCCCGTTTGACACAAAAGCAATCGAAGAGTTGTACGTAGAGTTAGCTTCTAGACGCTCTGAGTTGCTCCGTAAATTGACCGAAACGTTCGGCTCGTGGTATCAGCCTAAAGGTGGCACTGAGATGt                                                                                                                                                                                                                             <  1:522523/300‑1 (MQ=255)`  
                                                                             `ataagagacagcTAAAGCTCTCCTTGAGAAGCTACTCTCTGACAAACATTACTTCCCTCCTGAGATTGACTTTACGGACGTAGGATACACTACGTTCTGGTCAGAAT‑CCCTTGAGGCCGTTGACATTGTACATCGTGCTGCATGGCTGCTCGCTAAACAAGAGCGCAACGGGTTCCCGTTTGACACAAAAGCAATCGAAGAGTTGTACGTAGAGTTAGCTTCTCGCCGCTCTGAGTTGCTCCGTAAATTGACCGAAACGTTCGGATCGTGGTATCAGCCTAAAGGTGGCACTGAGATGTTc                                                                                                                                                                                                                           <  1:364277/290‑1 (MQ=255)`  
                                                                                 `gtgGTAACTAAAGCTCTCCTTGAGAAGCTACTCTCTGACAAACATTACTTCCCTCCTGAGATTGACTTTACGGACGTAGGATACACTACGTTCTGGTCAGAAT‑CCCTTGAGGCCGTTGACATTGAACATCGTGCTGCATGGCTGCTCGCTAAACAAGAGCGCAACGGGTTCCCGTTTGACACAAAAGCAATCGAAGAGTTGTACGTAGAGTTAGCTTCTCGCCGCTCTGAGTTGCTCCGTAAATTGACCGAAAAGTTCGGCTCGTGGTATCAGCCTAAAGGTGGCACTGAGATGTTCTGcc                                                                                                                                                                                                                       >  1:788113/1‑301 (MQ=255)`  
                                                                                      `aaCTAAAGCTCTCCTTGAGAAGCTACTCTCTGACAAACATTACTTCCCTCCTGAGATTGACTTTACGGACGTAGGATACACTACGTTCTGGTCAGAATCCCCTTGAGGCCGTTGACATTGAACATCGTGCTGCATGGCTGCTCGCTAAACAAGAGCGCAACGGGTTCCCGTTTGACACAAAAGCAATCGAAGAGTTGTACGTAGAGTTAGCTTCTCGCCGCTCTGAGTTGCTCCGTAAATTGACCGAAACGTTCGGCTCGTGGTATCAGCCTAAAGGTGGCACTGAGATGTTCTGCCATcc                                                                                                                                                                                                                   >  1:296973/1‑301 (MQ=255)`  
                                                                                      `aaCTAAAGCTCTCCTTGAGAAGCTACTCTCTGACAAACATTACTTCCCTCCTGAGATTGACTTTACGGACGTAGGATACACTACGTTCTGGTCAGAAT‑CCCTTGAGGCCGTTGACATTGAACATCGTGCTGCATGGCTGCTCGCTAAACAAGAGCGCAACGGGTTCCCGTTTGACACAAAAGCAATCGAAGAGTTGTACGTAGAGTTAGCTTCTCGCCGCTCTGAGTTGCTCCGTAAATTGACCGAAACGTTCGGCTCGTGGTATCAGCCTAAAGGTGGCACTGAGATGTTCTTCCATCcg                                                                                                                                                                                                                  >  1:604603/1‑301 (MQ=255)`  
                                                                                      `aaCTAAAGCTCTCCTTGAGAAGCTACTCTCTGACAAACATTACTTCCCTCCTGAGATTGACTTTACGGACGTAGGATACACTACGTTCTGGTCAGAAT‑CCCTTGAGGCCGTTGACATTGAACATCGTGCTGCATGGCTGCTCGCTAAACAAGAGCGCAACGGGTTCCCGTTTGACACAAAAGCAATCGAAGAGTTGTACGTAGAGTTAGCTTCTCGCCGCTCTGAGTTGCTCCGTAAATTGACCGAAACGTTCGGCTCGTGGTATCAGCCTAAAGGTGGCACTGAGATGTCCTGCCactgt                                                                                                                                                                                                                  >  1:131448/1‑297 (MQ=255)`  
                                                                                        `cTAAAGCTCTCCTTGAGAAGCTACTCTCTGACAAACATTACTTCCCTCCTGAGATTGACTTTACGGACGTAGGATACACTACGTTCTGGTCAGAAT‑CCCTTGAGGCCGTTGACATTGAACATCGTGCTGCATGGCTGCTCGCTAAACAAGAGCGCAACGGGTTCCCGTTTGACACAAAAGCAATCGAAGAGTTGTACGTAGAGTTAGCTTCTCGCCGCTCTGAGTTGCTCCGTAAATTGACCGAAACGTTCGGCTCGTGGTATCAGCCTAAAGGTGGCACTGAGATGTTCTGCCactgtct                                                                                                                                                                                                                >  1:571756/1‑295 (MQ=255)`  
                                                                                           `gacagctcCTTGAGAAGCTACTCTCTGACAAACATTACTTCCCTCCTGAGATTGACTTTACGGACGTAGGATACACTACGTTCTGGTCAGAAT‑CCCTTGAGGCCGTTGACATTGAACATCGTGCTGCATGGCTGCTCGCTAAACAAGAGCGCAACGGGTTCCCGTTTGACACAAAAGCAATCGAAGAGTTGTACGTAGAGTTAGCTTCTCGCCGCTCTGAGTTGCTCCGTAAATTGACCGAAACGTTCGGCTCGTGGTATCAGCCTAAAGGTGGCACTGAGATGTTCTGCCATCCGCGAAc                                                                                                                                                                                                             <  1:817235/296‑1 (MQ=255)`  
                                                                                                  `ccTTGAGAAGCTACTCTCTGACAAACATTACTTCCCTCCTGAGATTGACTTTACGGACGTAGGATACACTACGTTCTGGTCAGAAT‑CCCTTGAGGCCGTTGACATTGAACATCGTGCTGCATGGCTGCTCGCTAAACAAGAGCGCAACGGGTTCCCGTTTGACACAAAAGCAATCGAAGAGTTGTACGTAGAGTTAGCTTCTCGCCGCTCTGAGTTGCTCCGTAAATTGACCGAAACGTTCGGCTCGTGGTATCAGCCTAAAGGTGGCCCTGAGATGTTCTGCCATCCGCGAACAGGTAAg                                                                                                                                                                                                      >  1:542355/1‑301 (MQ=255)`  
                                                                                                         `aaGCTACTCTCTGACAAACATTACTTCCCTCCTGAGATTGACTTTACGGACGTAGGATACACTACGTTCTGGTCAGAAT‑CCCTTGAGGCCGTTGACATTGAACATCGTGCTGCATGGCTGCTCGCTAAACAAGAGCGCAACGGGTTCCCGTTTGACACAAAAGCAATCGAAGAGTTGTACGTAGAGTTAGCTTCTCGCCGCTCTGAGTTGCTCCGTAAATTGACCGAAACGTTCGGCTCGTGGTATCAGCCTAAAGGTGGCACTGAGATGTTCTGCCATCCGCGAACAGGTAAGCCACTAc                                                                                                                                                                                               >  1:195386/1‑301 (MQ=255)`  
                                                                                                            `cTACTCTCTGACAAACATTACGTCCCTCCTGAGATTGACTTTACGGACGTAGGATACACTACGTTCTGGTCAGAAT‑CCCTTGAGGCCGTTGACATTGAACATCTTGCTGCATGGCTGCTCGCTAAACAAGAGCGCAACGGGTTCCCGTTTGACACAAAAGCAAGCGAAGAGTTGTACGTAGAGTTAGCTTCTCGCCGCTCTGAGTTGCTCCGTAAATTGACCGAAACGTTCGGCTCGTGGTATCAGCCTAAAGGTGGCACTGAGATGTTCTTCCATACGCGAACAGGTAAGCCACTACCTa                                                                                                                                                                                            <  1:326782/301‑1 (MQ=255)`  
                                                                                                                `tctctGACAAACATTACTTCCCTCCTGAGATTGACTTTACGGACGTAGGATACACTACGTTCTGGTCAGAAT‑CCCTTGAGGCCGTTGACATTGAACATCGTGCTGCATGGCTGCTCGCTAAACAAGAGCGCAACGGGTTCCCGTTTGACACAAAAGCAATCGAAGAGTTGTACGTAGAGTTAGCTTCTCGCCGCTCTGAGTTGCTCCGTAAATTGACCGAAACGTTCGGCTCGTGGTATCAGCCTAAAGGTGGCACTGAGATGTTCTGCCATCCGCGAACAGGTAAGCCACTACCTAAATa                                                                                                                                                                                        >  1:781337/1‑301 (MQ=255)`  
                                                                                                                 `ctctGACAAACATTACTTCCCTCCTGAGATTGACTTTACGGACGTAGGATACACTACGTTCTGGTCAGAAT‑CCCTTGAGGCCGTTGACATTGAACATCGTGCTGCATGGCTGCTCGCTAAACAAGAGCGCAACGGGTTCCCGTTTGACACAAAAGCAATCGAAGAGTTGTACGTAGAGTTAGCTTCTCGCCGCTCTGAGTTGCTCCGTAAATTGACCGAAACGTTCGGCTCGTGGTATCAGCCTAAAGGTGGCACTGAGATGTTCTGCCATCCGCGAACAGGTAAGCCACTACCTAAATAc                                                                                                                                                                                       >  1:340941/1‑301 (MQ=255)`  
                                                                                                                 `ctctGACAAACATTACTTCCCTCCTGAGATTGACTTTACGGACGTAGGATACACTACGTTCTGGTCAGAAT‑CCCTTGAGGCCGTTGACATTGAACATCGTGCTGCATGGCTGCTCGCTAAACAAGAGCGCAACGGGTTCCCGTTTGACACAAAAGCAATCGAAGAGTTGTACGTAGAGTTAGCTTCTCGCCGCTCTGAGTTGCTCCGTAAATTGACCGAAACGTTCGGCTCGTGGTATCAGCCTAAAGGTGGCACTGAGATGTTCTGCCAGCCGCGAACAGGTAAGCCACTACCTAAATAc                                                                                                                                                                                       <  1:720684/301‑1 (MQ=255)`  
                                                                                                                 `cactGACAAACATTACTTCCCTCCTGAGATTGACTTTACGGACGTAGGATACACTACGTTCTGGTCAGAAT‑CCCTGGAGGCCGTTGACATTGAACATCGTGCTGCATGGCTGCTCGCTAAACAAGAGCGCAACGGGTTCCCGTTTGACACAAAAGCAATCGAAGAGTTGTACGTAGAGTTAGCTTCTCGCCGCTCTGAGTTGCTCCGTAAATTGACCGAAACGTTCGGCTCGTGGTATCAGCCTAAAGGTGGCACTGAGATGTTCTGCCATCCGCGAACAGGTAAGCCACTACCTAAATAc                                                                                                                                                                                       <  1:146463/299‑1 (MQ=255)`  
                                                                                                                 `aaagagacagcATTACTTCCCTCCTGAGATTGACTTTACGGACGTAGGATACACTACGTTCTGGTCAGAAT‑CCCTTGAGGCCGTTGACATTGAACATCGTGCTGCATGGCTGCTCGCTAAACAAGAGCGCAACGGGTTCCCGTTTGACACAAAAGCAATCGAAGAGTTGTACGTAGAGTTAGCTTCTCGCCGCTCTGAGTTGCTCCGTAAATTGACCGAAACGTTCGGCTCGTGGTATCAGCCTAAAGGTGGCACTGAGATGTTCTGCCATCCGCGAACAGGTAAGCCACTACCTAAATAc                                                                                                                                                                                       <  1:332750/291‑1 (MQ=255)`  
                                                                                                                       `cAAACATTACTTCCCTCCTGAGATTGACTTTACGGACGTAGGATACACTACGTTCTGGTCAGAAT‑CCCTTGAGGCCGTTGACATTGAACATCGTGCTGCATGGCTGCTCGCTAAACAAGAGCGCAACGGGTTCCCGTTTGACACAAAAGCAATCGAAGAGTTGTACGTAGAGTTAGCTTCTCGCCGCTCTGAGTTGCTCCGTAAATTGACCGAAACGTTCGGCTCGTGGTATCAGCCTAAAGGTGGCACTGAGATGTTCTGCCATCCGCGAACAGGTAAGCCACTACCTAAATACCCTCGc                                                                                                                                                                                 >  1:767005/1‑301 (MQ=255)`  
                                                                                                                         `aaCATTACTTCCCTCCTGAGATTGACTTTACGGACGTAGGATACACTACGTTCTGGTCAGAAT‑CCCTTGAGGCCGTTGACATTGAACATCGTGCTGCATGGCTGCTCGCTAAACAAGAGCGCAACGGGTTCCCGTTTGACACAAAAGCAATCGAAGAGTTGTACGTAGAGTTAGCTTCTCGCCGCTCTGAGTTGCTCCGTAAATTGACCGAAACGTTCGGCTCGTGGTATCAGCCTAAAGGTGGCACTTAGATGTTCTGCCATCCGCGAACAGGTAAGCCACTACCTAAATACCCTCGCAt                                                                                                                                                                               <  1:488310/301‑1 (MQ=255)`  
                                                                                                                           `cATTACTTCCCTCCTGAGATTGACTTTACGGACGTAGGATACACTACGTTCTGGTCAGAAT‑CCCTTGAGGACGTTGACATTGAACATCGTGCTGCATGGCTGCTCGCTAAACAAGAGCGCAACGGGTTCCCGTTTGACACAAAAGCAATCGAAGAGTTGTACGTAGAGTTAGCTTCTCGCCGCTCTGAGTTGCTCCGTAAATTGACCGAAACGTTCGGCTCGTGGTATCAGCCTAAAGGTGGCACTGAGATGTTCTGCCATCCGCGAACAGGTAAGCCACTACCTAAATACCCTCGCATTa                                                                                                                                                                             >  1:210398/1‑301 (MQ=255)`  
                                                                                                                               `gcgacagtccaGAGATTGGCTTTACGGACGTAGGATACACTACGTTCTGGTCAGAAT‑CCCTTGAGGCCGTTGACATTGGACATCGTGCTGAATGGCTGCTCGCTAAACAAGAGCGCAACGGGTTCACGTTTGACACAAAAGCAAGCGAAGAGTTGTACGTAGAGTTAGCTTCTCGCCGCTCTGAGTTGCTCCGTAAATTGACCGAAACGTTCGGCTCGTGGTATCAGCCTAAAGGTGGCACTGAGATGTTCTGCCATCCGCGAACAGGTAAGCCACTACCTAAATACCCTCGCATTAAGac                                                                                                                                                                         <  1:824484/294‑1 (MQ=255)`  
                                                                                                                               `ccTTCCCTCCTGAGATTGACTTTACGGACGTAGGATACACTACGTTCTGGTCAGAAT‑CCCTTGAGGCCGTTGACATTGAACATCGTGCTGCATGGCTGCTCGCTAAACAAGAGCGCAACGGGTTCCCGTTTGACACAAAAGCAATCGAAGAGTTGTACGTAGAGTTAGCTTCTCGCCGCTCTGAGTTGCTCCGTAAATTGACCGAAACGTTCGGCTCGTGGTATCAGCCTAAAGGTGGCACTGAGATGTTCTGCCATCCGCGAACAGGTAAGCCACTACCTAAATACCCTCGCATTAAGac                                                                                                                                                                         <  1:611727/300‑1 (MQ=255)`  
                                                                                                                                `cTTCCCTCCTGAGATTGACTTTACGGACGTAGGATACACTACGTTCTGGTCAGAAT‑CCCTTGAGGCCGTTGACATTGAACATCGTGCTGCATGGCTGCTCGCTAAACAAGAGCGCAACGGGTTCCCGTTTGACACAAAAGCAATCGAAGAGTTGTACGTAGAGTTAGCTTCTCGCCGCTCTGAGTTGCTCCGTAAATTGACCGAAACGTTCGGCTCGTGGTATCAGCCTAAAGGTGGCACTGAGATGTTCTGCCATCTGCGAACAGGTAAGCCACTACCTAAATAcctgtctcttatacac                                                                                                                                                                        >  1:372694/1‑287 (MQ=255)`  
                                                                                                                                       `aaagtgtataatagacaggACGTAGGATACTCTACGTTCGGGTCAGAAT‑CCCTTGAGTCCGTTGACATTGAACATCGTGCTGCATGGCTGCTCGCTAAACAAGTGCGCAACGGGTTCCCGTTTGACACAAAAGCAATCGAAGAGTTGTACGTAGAGTTAGCTACTCGCCGCTCTGAGTTGATCCGTAAATTGACCGAAACGTTCGGCTCGTGGTATCAGCCTAAAGGTTGCACTGAGATGTTCTGCCAGCCGCGAACAGGTAAGCCACTACCTAAATACCCTCGCATTAAGACACCTAAAg                                                                                                                                                                 <  1:239646/284‑1 (MQ=255)`  
                                                                                                                                        `tcggagatgtgtataagagacagggATACACTACGTTCTGGTCAGAAT‑CCCTTGAGGCCGTTGACATTGAACATCGTGCTGCATGGCTGCTCGCTAAACAAGAGCGCAATGGGTTCCCGTTTGACACAAAAGCAATCGAAGAGTTGTACGTAGAGTTAGCTTCTCGCCGCTCTGAGTTGCTCCGTAAATTGACCGAAACGTTCGGCTCGTGGTATCAGCCTAAAGGTGGCACTGAGATGTTCTGCCATCCGCGAACAGGTAAGCCACTACCTAAATACCCTCGCATTAAGACACCTAAAGt                                                                                                                                                                <  1:79629/278‑1 (MQ=255)`  
                                                                                                                                         `ggagatgtgtataagagacagaGGAAACACTACGTTCTGGTCAGAAT‑CCCTTGAGGCCGTTGACATTGAACATCGTGCTGCATGGCTGCTCGCTAAACAAGAGCGCAACGGGTTCCCGTTTGACACAAAAGCAATCGAAGAGTTGTACGTAGAGTTAGCTTCTCGCCGCTCTGAGTTGCTCCGTAAATTGACCGAAACGTTCGGCTCGTGGTATCAGCCTAAAGGTGGCACTGAGATGTTCTGCCATCCGCGAACAGGTAAGCCACTACCTAAATACCCTCGCATTAAGACACCTAAAGtt                                                                                                                                                               <  1:379819/280‑1 (MQ=255)`  
                                                                                                                                           `agaTTGACTTTACGGACGTAGGATACACTACGTTCTGGTCAGAAT‑CCCTTGAGGCCGTTGACATTGAACATCGTGCTGCATGGCTGCTCGCTAAACAAGAGCGCAACGGGTTCCCGTTTGACACAAAAGCAATCGAAGAGTTGTACGTAGAGTTAGCTTCTCGCCGCTCTGAGTTGCTCCGTAAATTGACCGAAACGTTCGGCTCGTGGTATCAGCCTAAAGGTGGCACTGAGATGTTCTGCCATCCGCGAACAGGTAAGCCACTACCTAAATACCCTCGCATTAAGACACCTAAAGTtgg                                                                                                                                                             <  1:676766/301‑1 (MQ=255)`  
                                                                                                                                            `gaTTGACTTTACGGACGTAGGATACACTACGTTCTGGTCAGAAT‑CCCTTGAGGCCGTTGACATTGAACATCGTGCTGCATGGCTGCTCGCTAAACAAGAGCGCAACGGGTTCCCGTTTGACACAAAAGCAATCGAAGAGTTGTACGTAGAGTTAGCTTCTCGCCGCTCTGAGTTGCTCCGTAAATTGACCGAAACGTTCGGCTCGTGGTATCAGCCTAAAGGTGGCACTGAGATGTTCTGCCATCCGCGAACAGGTAAGCCACTACCTAAATACCCCCGCATTAAGAAACCTAAAGTtgcg                                                                                                                                                            >  1:744330/1‑299 (MQ=255)`  
                                                                                                                                                  `aagacaggACGAAGGATAAAATACGTTCTGGTAAGAAT‑CCCATGAGGCAGTTGACATTGAACATCGTGCTGAATGGCTGCTCGCTAAACAAGAGCGCAACGGGTTCCCGTTAGACACAAAAGCAAACGAAGAGTTGTACGAAGAGTTAGCTTCAAGACGATCTGAGTTGCACCGTAAATTGAAATAAACGTTCGGATCGTGGTATCAGCCTAAAGGTGGAACTGAGATGTTCTGCCATCCGCGAACAGGTAAGCCACTACCTAAATACCCTCGCATTAAGACACCTAAAGTTGGTGGTATc                                                                                                                                                      <  1:304868/295‑1 (MQ=255)`  
                                                                                                                                                         `gACGTAGGATACACTACGTTCTGGTCAGAAT‑CCCTTGAGGCCGTTGACATTGAACATCGTGCTGCATGGCTGCTCGCTAAACAAGAGCGCAACGGGTTCCCGTTTGACACAAAAGCAATCGAAGAGTTGTACGTAGAGTTAGCTTCTCGCCGCTCTGAGTTGCTCCGTAAATTGACCGAAACGTTCGGCTCGTGGTATCAGCCTAAAGGTGGCACTGAGATGTTCTGCCATCCGCGAACAGGTAAGCCACTACCTAAATACCCTCGCATTAAGACACCTAAAGTTGGTGGTATCTTTaaga                                                                                                                                               >  1:189025/1‑301 (MQ=255)`  
                                                                                                                                                         `gACGTAGGATACACTACGTTCTGGTCAGAAT‑CCCTTGAGGCCGTTGACATTGAACATCGTGCTGCATGGCTGCTCGCTAAACAAGAGCGCAACGGGTTCCCGTTTGACACAAAAGCAATCGAAGAGTTGTACGTAGAGTTAGCTTCTCGCCGCTCTGAGTTGCTCCGTAAATTGACCGAAACGTTCGGCTCGTGGTATCAGCCTAAAGGTGGCACTGAGATGTTCTGCCATCCGCGAACAGGTAAGCCACTACCTAAATACCCTCACATTAAGACACCTAAAGCtgtctcttatacacatc                                                                                                                                               >  1:166262/1‑286 (MQ=255)`  
                                                                                                                                                          `cggagatgtgtataagagacaggTCAGAAT‑CCCTTGAGGCCGTTGACATTGAACATCGTGCTGCATGGCTGCTCTCTAAACAAGAGCGCAACGGGTTACCGTTTGACACAAAAGCAATCGAAGAGTTGTACGTAGAGTTAGCTTCTCGCCGCTCTGAGTTGCTCCGTAAATTGACCGAAACGTTCGGCTCGTGGTATCAGCCTAAAGGTGGCACTGAGATGTTCTGCCATCCGCGAACAGGTAAGCCACTACCTAAATACCCTCGCATTAAGACACCTAAAGTTGGTGGTATCTTTaagaa                                                                                                                                              <  1:16046/280‑1 (MQ=255)`  
                                                                                                                                                                    `cacTACTTTCTGGTCAGAAT‑CCCGTGAGGCCGTTGACATTGAACATCGTGCTGCATGGCTGCTCGCTAAACAAGAGCGAAACAGGTTCCCGTTAGACAAAAAAGCAATCGAAGAGTTGTACGTAGAGTTAGCTTCTCGCCGCTCTGAGTTGCTCCGTAAATTGACCGAAACGTTCGGCTCGTGGTATCAGCCTAAAGGTAGCACTGAGATGTTCTGCCATCCGCGAACAGGTAAGCCACTACCTAAATACCCTCGCATTAAGACACCTAAAGTTGGTGGTATCTTTAAGAAGCCTAAGAAc                                                                                                                                    <  1:139516/301‑1 (MQ=255)`  
                                                                                                                                                                    `cacTACGTTCTGGTCAGAAT‑CCCTTGAGGCCGTTGACATTGAACATCGTGCTGCATGGCTGCTCGCTAAACAAGAGCGCAACGGGTTCCCGTTTGACACAAAAGCAATCGAAGAGTTGTACGTAGAGTTAGCTTCTCGCCGCTCTGAGTTGCTCCGTAAATTGACCGAAACGTTCGGCTCGTGGTATCAGCCTAAAGGTGGCACTGAGATGTTCTGCCATCCGCGAACAGGTAATCCACTACCTAAATACCCTCGCATTAAGACACCTAAAGTTGGTGGTATCTTTAAGAAGCCTAAGAAc                                                                                                                                    <  1:410001/301‑1 (MQ=255)`  
                                                                                                                                                                    `cacTACGTTCTGGTCAGAAT‑CCCTTGAGGCCGTTGACATTGAACATCGTGCTGCATGGCTGCTCGCTAAACAAGAGCGCAACGGGTTCCCGTTTGACACAAAAGCAATCGAAGAGTTGTACGTAGAGTTAGCTTCTCGCCGCTCTGAGTTGCTCCGTAAATTGACCGAAACGTTCGGCTCGTGGTATCAGCCTAAAGGTGGCACTGAGATGTTCTGCCATCCGCGAACAGGTAAGCCACTACCTAAATACCCTCGCATTAAGACACCTACAGTTGGTGGTATCTTTAAGAAGCCTAAGAAc                                                                                                                                    >  1:474173/1‑301 (MQ=255)`  
                                                                                                                                                                    `cacTACGTTCTGGTCAGAAT‑CCCTTGAGGCCGTTGACATTGAACATCGTGCTGCATGGCTGCTCGCTAAACAAGAGCGCAACGGGTTCCCGTTTGACACAAAAGCAATCGAAGAGTTGTACGTAGAGTTAGCTTCTCGCCGCTCTGAGTTGCTCCGTAAATTGACCGAAACGTTCGGCTCGTGGTATCAGCCTAAAGGTGGCACTGAGATGTTCTGCCATCCGCGAACAGGTAAGCCACTACCTAAATACCCTCGCATTAAGACACCTAAAGTTGGTGGTATCTTTAAGAAGCCTAAGAAc                                                                                                                                    >  1:579746/1‑301 (MQ=255)`  
                                                                                                                                                                    `cacTACGTTCTGGTCAGAAT‑CCCTTGAGGCCGTTGACATTGAACATCGTGCTGCATGGCTGCTCGCTAAACAAGAGCGCAACGGGTTCCCGTTTGACACAAAAGCAATCGAAGAGTTGTACGTAGAGTTAGCTTCTCGCCGCTCTGAGTTGCTCCGTAAATTGACCGAAACGTTCGGCTCGTGGTATCAGCCTAAAGGTGGCACTGAGATGTTCTGCCATCCGCGAACAGGTAAGCCACTACCTAAATACCCTCGCATTAAGACACCTAAAGTTGGTGGTATCTTTAAGAAGCCTAAGAAc                                                                                                                                    >  1:801755/1‑301 (MQ=255)`  
                                                                                                                                                                    `cacTACGTTCTGGTCAGAAT‑CCCTTGAGGCCGTTGACATTGAACATCGTGCTGCATGGCTGCTCGCTAAACAAGAGCGCAACGGGTTCCCGTTTGACACAAAAGCAATCGAAGAGTTGTACGTAGAGTTAGCTTCTCGCCGCTCTGAGTTGCTCCGTAAATTGACCGAAACGTTCGGCTCGTGGTATCAGCCTAAAGGTGGCACTGAGATGTTCTGCCATCCGCGAACAGGTAAGCCACTACCTAAATACCCTCGCATTAAGACACCTAAAGTTGGTGGTATCTTTAAGAAGCCTAAGAAc                                                                                                                                    >  1:253088/1‑301 (MQ=255)`  
                                                                                                                                                                    `aacTACGTTCTGGTCAGAAT‑CACTTGAGGCCGTTGACAGTGAACATCGTGCTGCATGGCTGCGCGCTAAACAAGAGCGCAACGGGTTCCCGTTTGACACAAAAGCAATCGAAGAGTTGTACGTAGAGTTAGCTTCTCGCCGCTCTGAGTTGCTCCGTAAATTGACCGAAACGTTCGGCTCGTGGTATCAGCCTAAAGGTGGCACTGAGATGTTCTGCCATCCGCGAACAGGTAAGCCACTACCTAAATACCCTCGCATTAAGACACCTAAAGTTGGTGGTATCTTTAAGAAGCCTAAGAAc                                                                                                                                    <  1:356284/300‑1 (MQ=255)`  
                                                                                                                                                                    `aaatACGTTCTGGTCAGAAT‑CCCTTGAGGACGTTGACATTGAACATCGTGCTGCATGGCTGCTCGCTAAACAAGAGCGCAACGGGTTCCCGTTTGACACAAAAGCAATCGAAGAGTTGTACGTAGAGTTAGCTTCTCGACGCTCTGAGTTGCTCCGTAAATTGTCCGAAACGTTCGGCTCGTGGTATCAGCCTAAAGGTGGCACTGAGATGTTCTGCCATCCGAGAACAGGTAAGCCACTACCTAAATACCCTCGCATTAAGACACCTAAAGTTGGTGGTATCTTTAAGAAGCCTAAGAAc                                                                                                                                    <  1:418436/298‑1 (MQ=255)`  
                                                                                                                                                                      `cTACGTTCTGGTCAGAAT‑CCCTTGAGGCCGTTGACATTGAACATCGTGCTGCATGGCTGCTCGCTAAACAAGAGCGCCACGGGTTCCCGTTTGACACAAAAGCAATCGAAGCGTTGTACGTAGAGTTAGCTTCTCGCCGCTCTGAGTTGCTCCGTAAATTGACCGAAACGTTCGGCTCGTGGTATCAGCCTAAAGGTGGCACTGAGATGTTCTGCCATCCGCGCACAGGTAAGCCACTACCTAAATACCCTCGCATTAAGACACCTCAAGTTGGTGGTATCTTTAAGAAGTCTACGAACaa                                                                                                                                  >  1:92413/1‑301 (MQ=255)`  
                                                                                                                                                                         `cGTTCAGGTCAGAAT‑CCCTTGAGGCCGTTGACATTGAACATCGTGCTGCATGGCTGCTCGCTAAACAAGAGCGCAACGGGTTCCCGTTTGACACAAAAGCAATCGAAGAGTTGTACGTAGAGTTAGCTTCTCGCCGCTCTGAGTTGCTCCGTAAATTGACCGAAACGTTCGGCTCGTGGTATCAGCCTAAAGGTGGCACTGAGATGTTCTGCCATCCGCGAACAGGTAAGCCACTACCTAAATACCCTCGCATTAAGACACCTAAAGTTGGTGGTATCTTTAAGAAGCCTAAGAACAAGGc                                                                                                                               <  1:39521/301‑1 (MQ=255)`  
                                                                                                                                                                                       `gggctcggagatgtgtataagagacagtCGAGCTGCATGGCTGCTCGCTAAACAAGAGCGCAACGGGTTCCCGTTTGACACAAAAGCAATCGAAGAGTTGTACGTATAGTTAGCTTCTCGCCGCTCTGAGTTGCTCCGTAAATTGACCGAAACGTTCGGCTCGTGGTATCAGCCTAAAGGTGGCACTGAGATGTTCTGCCATCCGCGAACAGGTAAGCCACTAACTAAATACCCTCGCATTAAGACACCTAAAGTTGGTGGTATCTTTAAGAAGCCTAAGAACAAGGCACAGCGAGAAGGc                                                                                                                  <  1:246470/274‑1 (MQ=255)`  
                                                                                                                                                                                         `cccTTGAGTCCGTTGACATTGAACATCGTGCTGCATGGCTGCTCGCTAAACAAGAGCGCAACGGGTTCCCGTTTGACACAAAAGCAATCGAAGAGTTGTACGTAGAGTTAGCTTCTCGCCACTCTGAGTTGCTCCGTAAATTGACCGAAACGTTCGGCTCGTGGTATCAGCCTAAAGGTAGCACTGAGATGTTCTGCCATCCGCGAACAGGTAAGCCACTACCTAAATACCCTCGCATTAAGACACCTAAAGTTGGTGGTATCTTTAAGAAGCCTAAGAACAAGGCACAGCTAGAAGGCCg                                                                                                                >  1:282408/1‑301 (MQ=255)`  
                                                                                                                                                                                           `cTTGAGGCCGTTGACATTGAACATCGTGCTGCATGGCTGCTCGCTAAACAAGAGCGCAACGGGTTCCCGTTTGACACAAAAGCAATCGAAGAGTTGTACGTAGAGTTAGCTTCTCGCCGCTCTGAGTTGCTCCGTAAATTGACCGAAACGTTCGGCTCGTGGTATCAGCCTAAAGGTGGCACTGAGATGTTCTGCCATCCGCGAACAGGTAAGCCACTACCTAAATACCCTCGCATTAAGACACCTAAAGTTGGTGGTATCTTTAAGAAGCCTAAGAACAAGGCACAGCGAGAAGGCCGTg                                                                                                              >  1:587056/1‑301 (MQ=255)`  
                                                                                                                                                                                                         `cATTGAACATCGTGCTGCATGGCTGCTCGCTAAACAAGAGCGCAACGGGTTCCCATTTGACACCAAAGCAATCGAAGAGTTGTACGTAGAGTTCGCTTCTCGCCGCTCTGAGTTGCTCCGTAAATTGACCGAAACGTTCGGCTCGTGGTATCAGCCTAAAGGTGGCACTGAGATGTTCTGCCATCCGCGAACAGGTAAGCCACTACCTACATACCCTCGCATTAAGACACCTAAAGTTGGTGGTATCTTTAAGAAGCCTAAGAACAAGGCACAGCGAGAAGGCCGTGAGCCtgtctcttat                                                                                                >  1:51474/1‑292 (MQ=255)`  
                                                                                                                                                                                                               `aCATCGTGCTGCATGGCTGCTCGCTAAACAAGAGCTCAACGGGTTCCCGTTTGACACAAAAGCAATCGAAGAGTTGTACGTAGAGTTAGCTTCTCGCCGCTCTGAGTTGCTCCGTAAATTGACCGAAACGTTCGGCTCGTGGTATCAGCCTAAAGGTGGCACTGAGATGTTCTGCCATCCGCGAACAGGTAAGCCACTACCTAAATACCCTCGCATTAAGACACCTAAAGTTGGTGGTATCTTTAAGAAGCCTAAGAACAAGGCACAGCGAGAAGGCCGTGAGCCTTGCGAACTTGATAcc                                                                                          <  1:647890/301‑1 (MQ=255)`  
                                                                                                                                                                                                               `aCATCGTGCTGCATGGCTGCTCGCTAAACAAGAGCGCAACGGGTTCCCGTTTGACACAAAAGCAATCGAAGAGTTGTACGTAGAGTTAGCTTCTCGCCGCTCTGAGTTGCTCCGTAAATTGACCGAAACGTTCGGCTCGTGGTATCAGCCTAAAGGTGGCACTGAGATGTTCTGCCATCCGCGAACAGGTAAGCCACTACCTAAATACCCTCGCATTAAGACACCTAAAGTTGGTGGTATCTTTAAGAAGCCTAAGAACAAGGCACAGCGAGAAGGCCGTGAGCCTTGCGAACTTGATAcc                                                                                          <  1:203799/301‑1 (MQ=255)`  
                                                                                                                                                                                                               `aCATCGTGCTGCATGGCGGCTCACTAAACAAGAGCGAAACGGGGTCCCGTTTGACACAAAAGCAATCGAAGAGTTGTACGTAGAGTTAGCTTCTCGCAGCTCTGAGTTGCTCCGTAAATTGACCGAAACGGTCGGCTCGTGGTAGCAGCCTAAAGGTGGCACTGAGATGTTCTGCCATCCGCGAACAGGTAAGCCACTACCTAAATACCCTCGCATTAAGACACCTAAAGTTGGTGGTATCTTTAAGAAGCCTAAGAACAAGGCACAGCGAGTAGGCCGTGAGCCTTGCGAACTTGATAcc                                                                                          <  1:427840/301‑1 (MQ=255)`  
                                                                                                                                                                                                               `aCATCGTGCTGAATGGCTGATCGCTAAACAAGAGCGCAACGGGTTCCCGTTTGACACAAAAGCAATCGAAGAGTTGTACGTAGAGTTAGCTTCTCGCCGCTCTGAGTTGCTCCGTAAATTGACCGAAGCGTTCGGCTCGTGGTATCAGCCTAAAGGTGGCACTGAGATGTTCTGCCATCCGCGAACAGGTAAGCCACTACCTAAATACCCTCGCATTAAGACACCTAAAGTTGGTGGTATCTTTATGAAGCCTAAGAACAAGGCACAGCGAGAAGGCCGTGAGCCTTGCGAACTTGATAcc                                                                                          <  1:655644/301‑1 (MQ=255)`  
                                                                                                                                                                                                                          `cATGGCTGCTCGCTAAACAAGAGCGCAACGGGTTCCCGTTTGACACAAAAGCAAGCGAAGAGTTGTACGTAGAGTTAGCTTCTCGCCGCTCTGAGTTGCTCCGTAAATTGACCGAAACGTTCGGCTCGTGGTATCAGCCTAAAGGTGGCACTGAGATGTTCTGCCATCCGCGAACAGGTAAGCCACTACCTAAATACCCTCGCATTAAGACACCTAAAGTTGGTGGTATCTTTAAGAAGCCTAAGAACAAGGCACAGCGAGAAGGCCGTGAGCCTTGCGAACTTGATACCCGCGAGTACGt                                                                               <  1:773393/301‑1 (MQ=255)`  
                                                                                                                                                                                                                                             `agagCGCAACGGTTTCCCGTTTGACACAAAAGCAATCGAAGATTTGTACGTAGAGTTAGCTTCTCGCCGCTCTGAGTTGCTCCGTAAATTGACCGAAACGTTCGGCTCGTGGTATCAGCCTAAACGTGGCACTTAGATGTTCTGCCATCCGCGAACAGGTAAGCCACTACCTAAATACCCTCGCATTAAGACACCTAAAGTTGGTGGTATCTTTAAGAAGCCTAAGAACAAGGCACAGCGAGAAGGCCGTGAGCCTTGCGAACTTGATACCCGCGAGTACGTTGCTGGTGCTCCTTACAcc                                                            <  1:259432/301‑1 (MQ=255)`  
                                                                                                                                                                                                                                             `agagCGCAACGGGTTCCCGTTTGACACAAAAGCAATCGAAGAGTTGTACGTAGAGTTAGCTTCTCGCCGCTCTGAGTTGCTCCGTAAATTGACCGAAACGTTCGGCTCGTGGTATCAGCCTAAAGGTGGCACTGAGATGTTCTGCCATCCGCGAACAGGTAAGCCACTACCTAAATACCCTCGCATTAAGACACCTAAAGTTGGTGGTATCTTTAAGAAGCCTAAGAACAAGGCACAGCGAGAAGGCCGTGAGCCTTGCGAACTTGATACCCGCGAGTACGTTGCTGGTGCTCCTTACAcc                                                            <  1:45861/301‑1 (MQ=255)`  
                                                                                                                                                                                                                                             `agagCGCAACGGGTGCCCGGTTGACACAAAAGCAATCGAAGAGTTGTACGTAGAGTTAGCTTCTCGCCGCTCTGAGTTGCTCCGTAAATTGACCGAAACGTTCGGCTCGTGGTATCAGCCTAAAGGTGGCACTGAGATGTTCTGCCATCCGCGAACAGGTAAGCCACTACCTAAATACCCTCGCATTAAGACACCTAAAGTTGGTGGTATCTTTAAGAAGCCTAAGAACAAGGCACAGCGAGAAGGCCGTGAGCCTTGCGAACTTGATACCCGCGAGTACGTTGCTGGTGCTCCTTACAcc                                                            <  1:267073/301‑1 (MQ=255)`  
                                                                                                                                                                                                                                              `gagCGCAACGGGTTCCCGTTTGACACAAAAGCAATCGAAGAGTTGTACGTAGAGTTAGCTTCTCGCCGCTCTGAGTTGCTCCGTAAATTGACAGAAACGTTCGGCTCGTGGTATCAGCCTAAAGGTTGCACTGAGATGTTCTGCCATCCGCGATCAGGTACGCCACTACCTAAATACCCTCGCATTAAGACACCTAAAGTTGGTGGTATCTTTAAGAAGCCTAAGAACAAGGCACAGCGAGAAGGCCGTGAGCCTTGCGAACTTGATACCCGCGAGTACGTTGCTGGTGCCCCTTACAccc                                                           >  1:810373/1‑301 (MQ=255)`  
                                                                                                                                                                                                                                                       `tgtataagagacagcacaAAAGCAATCGAAGAGTTGTACGTAGAGTTAGCTTCTCGCCGCTCTGAGTTGCTCCGTAAATTGACCGAAACGTTCGGCTCGTGGTATCAGCCTAAAGGTGGCACTGAGATGTTCTGCCATCCGCGAACAGGTAAGCCACTACCTAAATACCCTCGCATTAAGACACCTAAAGTTGGTGGTATCTTTAAGAAGCCTAAGAACAAGGCACAGCGAGAAGGCCGTGAGCCTTGCGAACTTGATACCCGCGAGTACGTTGCTGGTGCTCCTTACACCCCAGTTGAAc                                                  <  1:183105/287‑1 (MQ=255)`  
                                                                                                                                                                                                                                                         `tttCCCGTTTGACACAAAAGCAATCGAAGAGTTGTACGTAGAGTTAGCTTCTCGCCGCTCTGAGTTGCTCCGTAAATTGACCGAAACGTTCGGCTCGTGGTATCAGCCTAAAGGTGGCACTGAGATGTTCTGCCATCCGCGAACAGGTAAGCCACTACCTAAATACCCTCGCATTAAGACACCTAAAGTTGGTGGTATCTTTAAGAAGCCTAAGAACAAGGCACAGCGAGAAGGCCGTGAGCCTTGCGAACTTGATACCCGCGAGTACGTTGCTGGTGCTCCTTACACCCCAGTTGAACAt                                                <  1:348731/300‑1 (MQ=255)`  
                                                                                                                                                                                                                                                                    `acacAAAAGCAATCGAAGAGTTGTACGTAGAGTTAGCTTCTCGCCGCTCTGAGTTGCTCCGTAAATTGACCGAAACGTTCGGCTCGTGGTATCAGCCTAAAGGTGGCACTGAGATGTTCTGCCATCCGCGAACAGGTAAGCCACTACCTAAATACCCTCGCATTAAGACACCTAAAGTTGGTGGTATCTTTAAGAAGCCTAAGAACAAGGCACAGCGAGAAGGCCGTGAGCCTTGCGAACTTGATACCCGCGAGTACGTTGCTGGTGCTCCTTACACCCCAGTTGAACATGTTGTGTTTaa                                     >  1:85224/1‑301 (MQ=255)`  
                                                                                                                                                                                                                                                                       `aaaaaGCAATCGAATAGTTGTACGTAGAGTTAGCTTCTCGCCGCTCTGGGTTCCTCCGAAAATTGACCGAAACGTTCGGCTCGTGGTATCAGCCTAAAGGTGGCACTGAGATGTGCTGCCATCCGCGAACAGGTAAGCGACTACCTAAATACCCTCGCATTAAGACACCAAAAGTTGGTGGTATCTTTAAGAAGCCTAAGAACAAGGCACAGCGAGAAGGCCGTGAGCCTTGCGAACTTGATACCCGCGAGTACGTTGCTGGTGCTCCTTACACCCCAGTTGAACATGTTGTGTTTAAacc                                  <  1:217950/300‑1 (MQ=255)`  
                                                                                                                                                                                                                                                                                    `agatgtgtataagagacaggcttctCGCCGCTCTGAGTTGCTCCGTAAATTGACCGAAACGTTCGGCTCGTGGTATCAGCCTAAAGGTGGCACTGAGAAGTTCTGCCATCCGCGAACAGGTAAGCCACTACCTAAATACCCTCGCATTAAGACACCTAAAGTTGGTGGTATCTTTAAGAAGCCTAAGAACAAGGCACAGCGAGAAGGCCGTGAGCCTTGCGAACTTGATACCCGCGAGTACGTTGCTGGTACTCCTTACACCCCAGTTGAACATGTTGTGTTTAACCCTTCGTCTCATGAc                     <  1:40291/282‑1 (MQ=255)`  
                                                                                                                                                                                                                                                                                          `gTACGTAGAGTTAGCTTCTCGCCGCTCTGAGTTGCTCCGTAAATTGACCGAAACGTTCGGCTCGTGGTATCAGCCTAAAGGTGGCACTGAGATGTTCTGCCATCCGCGAACAGGTAAGCCACTACCTAAATACCCTCGCATTAAGACACCTAAAGTTGGTGGTATCTTTAAGAAGCCTAAGAACAAGGCACAGCGAGAAGGCCGTGAGCCTTGCGAACTTGATACCCGCGAGTACGTTGCTGGTGCTCCTTACACCCCAGTTGAACATGTTGTGTTTAACCCTTCGTCTCGTGACCACAtt               >  1:528625/1‑301 (MQ=255)`  
                                                                                                                                                                                                                                                                                          `gTACGTAGAGTTAGCTTCTCGCCGCTCTGAGTTGCTCCGTAAATTGACCGAAACGTTCGGCTCGTGGTATCAGCCTAAAGGTGGCACTGAGATGTTCTGCCATCCGCGAACAGGTAAGCCACTACCTAAATACCCTCGCATTAAGACACCTAAAGTTGGTGGTATCTTTAAGAAGCCTAAGAACAAGGCACAGCGAGAAGGCCGTGAGCCTTGCGAACTTGATACCCGCGAGTACGTTGCTGGTGCCCCTTACACCCCAGTTGAACATGTTGTGTTTAACCCTTCGTCTCGTGACTACAtt               >  1:682697/1‑301 (MQ=255)`  

                                                                                                                                                                                                                                                                                                          `|`                                                                                                                                                                                                                                                                                                           
`GAGCAGGGTGAAGAATACGTTGACGGAATGGAGTGGTGGAACTTCAACGAAGAGATGATGGACTATAACGTTCAGGACGTTGTGGTAACTAAAGCTCTCCTTGAGAAGCTACTCTCTGACAAACATTACTTCCCTCCTGAGATTGACTTTACGGACGTAGGATACACTACGTTCTGGTCAGAAT‑CCCTTGAGGCCGTTGACATTGAACATCGTGCTGCATGGCTGCTCGCTAAACAAGAGCGCAACGGGTTCCCGTTTGACACAAAAGCAATCGAAGAGTTGTACGTAGAGTTAGCTGCTCGCCGCTCTGAGTTGCTCCGTAAATTGACCGAAACGTTCGGCTCGTGGTATCAGCCTAAAGGTGGCACTGAGATGTTCTGCCATCCGCGAACAGGTAAGCCACTACCTAAATACCCTCGCATTAAGACACCTAAAGTTGGTGGTATCTTTAAGAAGCCTAAGAACAAGGCACAGCGAGAAGGCCGTGAGCCTTGCGAACTTGATACCCGCGAGTACGTTGCTGGTGCTCCTTACACCCCAGTTGAACATGTTGTGTTTAACCCTTCGTCTCGTGACCACATTCAGAAGAAACTCC  >  NC_001604.1/14797‑15391`

  

Alignment Legend

Aligned base mismatch/match (shaded by quality score): `ATCG/ATCG` `< 3 ≤` `ATCG/ATCG` `< 11 ≤` `ATCG/ATCG` `< 25 ≤` `ATCG/ATCG` `< 36 ≤` `ATCG/ATCG` `< 38 ≤` `ATCG/ATCG`

Unaligned base: `atcg`    Masked matching base: `atcg`    Alignment gap: `‑`    Deleted base: `‑`
